# Cecal motility and the impact of *Lactobacillus* in feather pecking laying hens

**DOI:** 10.1038/s41598-020-69928-6

**Published:** 2020-07-31

**Authors:** Nienke van Staaveren, Julia Krumma, Paul Forsythe, Joergen B. Kjaer, Isabelle Y. Kwon, Yu-Kang Mao, Christine West, Wolfgang Kunze, Alexandra Harlander-Matauschek

**Affiliations:** 1 Department of Animal Biosciences, University of Guelph, Guelph, ON Canada; 2 McMaster Brain-Body Institute, St. Joseph’s Healthcare, Hamilton, ON Canada; 3 Department of Medicine, McMaster University, Hamilton, ON Canada; 4 Firestone Institute for Respiratory Health, St. Joseph’s Healthcare, Hamilton, ON Canada; 5 Federal Research Institute for Animal Health, Institute of Animal Welfare and Animal Husbandry, Friedrich-Loeffler-Institut, Celle, Lower Saxony Germany; 6 Department of Biology, McMaster University, Hamilton, ON Canada; 7 Department of Psychiatry and Behavioural Neuroscience, McMaster University, Hamilton, ON Canada

**Keywords:** Animal physiology, Animal behaviour

## Abstract

The gut-microbiota-brain axis is implicated in the development of behavioural disorders in mammals. As such, its potential role in disruptive feather pecking (FP) in birds cannot be ignored. Birds with a higher propensity to perform FP have distinct microbiota profiles and feed transit times compared to non-pecking counterparts. Consequently, we hypothesize that the gut microbiota is intimately linked to FP and gut motility, which presents the possibility of using probiotics to control FP behaviour. In the present study, we aim to assess the relationship between cecal motility and the probiotic *Lactobacillus rhamnosus* in chickens classified as peckers (P, 13 birds) and non-peckers (NP, 17 birds). We show that cecal contractions were 68% less frequent and their amplitude increased by 58% in the presence of *L. rhamnosus*. Furthermore, the number of FP bouts performed by P birds was positively correlated with contraction velocity and amplitude. We present the first account of gut motility measurements in birds with distinct FP phenotypes. Importantly, the present work demonstrates the clear impact of a probiotic on cecal contractions. These findings lay the foundation for identifying biological differences between P and NP birds which will support the development of FP control strategies.

## Introduction

Veterinarians are routinely presented with birds affected by feather loss due to self-induced or bird-to-bird pecking^1^. In fact, feather pecking (FP) was listed as the most common behavioural issue in companion birds by 82% of veterinarians in the US, whereby 88% of veterinarians reported seeing more than one case per month^2^. Similar behaviour occurs in millions of domestic birds (*Gallus gallus domesticus*) kept for egg-laying in a variety of housing systems—from birds exclusively housed indoors to backyard chickens^3–5^. Risk factors for this behaviour in psittacines and other domestic birds can be medical, environmental, nutritional, and psychological in origin, but its multifactorial nature makes it difficult to treat^1^.

FP in domesticated birds is often thought to be a result of frustration in nutritionally, physically and sensorially deprived environments that prevent birds from performing highly motivated behaviour^6^. However, it is noteworthy that FP is also reported in birds having access to pasture where they are free and able to perform these same highly motivated behaviours^4,7,8^, suggesting that mechanisms other than inadequate environments may be at play. Interestingly, FP is associated with a range of neurological comorbidities, such as higher levels of fearfulness^9^, higher HPA-axis reactivity (i.e., interaction between the hypothalamus, pituitary gland, and the adrenal glands)^10^, and higher locomotor activity levels^11^. Additionally, upstream markers such as neurotransmitters and their precursors in the monoaminergic system, which are implicated in some neurobiological diseases, have been linked to FP behaviour^9,12–16^. Of the multiple comorbidities associated with FP, the role of the gastrointestinal (GI) conditions and the GI-brain axis in the development of FP has garnered increased attention in recent years^17^. For example, Birkl et al.^18^ and van der Eijk et al.^19^ showed that the cecal content of FP birds had a higher abundance of *Clostridiales* and a lower abundance of *Lactobacillus* compared to non-FP birds. The feed passage time through the GI tract of feather peckers is also faster compared to non-peckers^20^. Finally, these two groups of birds have been demonstrated to harbour distinct intestinal microbiota and short-chain fatty acid (SFCA) profiles^21,22^.

In humans, certain behavioural disorders are accompanied by changes in GI motility^23–27^. The GI microbiota and their fermentation products (e.g., SCFAs), the immune system, the central nervous system and the enteric nervous system (ENS) exert varying levels of control over GI motility. Furthermore, the aforementioned systems are interrelated, and so, disruption of any one system can cause GI motility alterations^28^. For instance, a mouse study showed that hypoplasia of the ENS led to slow GI transit times and reduced peristaltic reflex activity^29^. Pharmacological silencing of sensory neurons within the ENS also abolished propulsive peristalsis in the mouse intestine^30^. Furthermore, administration of *Lactobacillus reuteri* or *Lactobacillus rhamnosus* cultures is known to alter neural depended-GI motility reflexes by increasing the excitability of myenteric neurons, and thereby, altering vagal signaling from the GI tract to the brain^30–32^. Vagal afferent input to the brain leads to subsequent changes in brain chemistry, altering fear- and anxiety-related behaviour^33,34^. These previous studies demonstrate that microbial activity can modulate the excitability of the ENS by a rapid, drug-like action, but that it can also correct GI dysmotility and impact behaviour in rodents^33,35–37^. Whether GI disturbances merely contribute to core behavioural symptoms or whether they are the underlying cause of the latter is still unknown. Nevertheless, the use of so-called ‘beneficial’ GI microbiota to tackle behavioural and GI disorders is a prominent field of research in human and murine models^38^. Similarly, modulation of GI microbiota populations is suggested as an opportunity to improve the health of commercial poultry^39^.

Mirroring the mammalian models, we postulate that the GI microbiota exerts significant control over the avian ENS, altering intestinal motility, initiating signalling via the vagus nerve and, subsequently, impacting brain function. As a result, changes to the microbiota may alter FP behaviour in domesticated birds. In the present study, our first goal was to establish an ex-vivo model to study intestinal motility in avian subjects by examining excised GI tissue in an organ bath, similar to previously published murine studies^35–37,40^. Secondly, we investigated whether a probiotic treatment would impact motility measures in birds classified as feather peckers (P) or non-peckers (NP) using this established model. To this end, we first measured ENS-dependent propulsive peristalsis in P and NP birds by comparing the velocity, frequency and amplitude of cecal contractions. Subsequently, *Lactobacillus rhamnosus (JB-1)* was added to the cecal tissue as an intraluminal microbial stimulus. The choice of the beneficial bacteria as a stimulus was based on the combined findings that P birds had a lower abundance of *Lactobacillus*^18,19^ and an altered GI transit time^20^ compared to NP birds. Furthermore, *L. rhamnosus JB-1* had already been demonstrated to reverse stress-induced GI dysmotility in mice ^35,36^.

## Results

In total, 29 out of 30 birds showed contractions of one or both ceca during the experiment (Table 1). Both ceca contracted in 53% of the birds, while only one cecum contracted in 43% of the birds (Table 1). In cases where only once cecum contracted, the contraction was observed in the first cecum tested in 6 birds and it was observed in the second cecum in 7 birds. Out of the 60 tested ceca, 45 ceca were viable, and 37 and 33 of these ceca showed contractions during baseline and *L. rhamnosus* recordings, respectively. We found no statistical differences in velocity (F_1,15_ = 2.85, *P* = 0.1123), frequency (F_1,15_ = 0.28, *P* = 0.6054) and amplitude (F_1,11_ = 0.24, *P* = 0.6349) of contractions between the ceca of the birds (Table 2).

**Table 1:** Number of birds (P: peckers, NP: non-peckers) included in the experiment and number of birds with 0, 1, or 2 ceca showing contractions in the organ bath recordings.

	Total	P	NP
**No. of birds**	30	13	17
0 ceca	1	1	0
1 ceca	13	7	6
2 ceca	16	5	11

**Table 2:** Average velocity, frequency, and amplitude of cecal contractions as measured in the paired ceca (ceca 1 and ceca 2) in laying hens in an organ bath when perfused with baseline Krebs solution.

	Ceca 1	Ceca 2
Velocity (cm/s)	0.21 ± 0.04	0.28 ± 0.04
Frequency (Hz)	0.06 ± 0.008	0.06 ± 0.008
Amplitude (cm)	0.05 ± 0.009	0.05 ± 0.008

A total of 16 birds had two viable ceca that could be included in this comparison.

### Effect of intraluminal stimuli and FP phenotype on cecal contractions

No significant interactions between the FP phenotype and intraluminal stimuli (baseline vs *L. rhamnosus*) were observed for velocity (F_1,25_ = 0.24, *P* = 0.6264), frequency (F_1,25_ = 1.65, *P* = 0.2108), or amplitude (F_1,17_ = 2.07, *P* = 0.1679) of cecal contractions. Similarly, cecal contractions in P and NP birds had a similar velocity, frequency, and amplitude (Table 3).

**Table 3:** Average velocity, frequency, and amplitude of cecal contractions in laying hens as measured in an organ bath according to their feather pecking (FP) phenotype (peckers: P, non-peckers: NP) and intraluminal stimuli treatment (Baseline: Krebs solution, *L. rhamnosus: L. rhamnosus JB-1* dissolved in Krebs solution).

	FP phenotype			Luminal stimuli		
	P birds	NP birds	*P* value	Baseline	*L. rhamnosus*	*P* value
Velocity (cm/s)	0.22 ± 0.04	0.26 ± 0.03	0.3958	0.24 ± 0.03	0.24 ± 0.03	0.9898
Frequency (Hz)	0.04 ± 0.006	0.04 ± 0.006	0.8049	0.06 ± 0.006	0.02 ± 0.006	< 0.001
Amplitude (cm)	0.05 ± 0.010	0.06 ± 0.010	0.4895	0.04 ± 0.007	0.07 ± 0.012	0.0579

Interestingly, the comparison of intraluminal stimuli did reveal differences in measures of cecal contractions (Table 3). When the cecal tissue lumen was perfused with *L. rhamnosus* the frequency of contractions was 68% lower (Table 3), while the amplitude of contractions tended to be 59% higher (Table 3) compared to the baseline treatment as the intraluminal stimulus. Velocity was similar under the baseline and the *L. rhamnosus* treatments (Table 3).

### Correlations between FP behaviour and cecal motility

Relationships between FP behaviour and motility measures were further explored (Table 4). Positive correlations were found between the number of FP bouts performed and velocity (r = 0.72, *P* = 0.0123) and frequency (r = 0.66, *P* = 0.0267) of contractions in P birds, but no correlations were found in NP birds (Table 4).

**Table 4:** Spearman rank correlations between velocity (cm/s), frequency (Hz), and amplitude (cm) of cecal contractions as measured in an organ bath when perfused with baseline treatment (Krebs solution) and the average number of pecking bouts performed at the feather cover of laying hens in birds classed as peckers (P, n = 11) or non-peckers (NP, n = 13).

	P	NP
Velocity (cm/s)	0.72*	− 0.40
Frequency (Hz)	− 0.02	0.006
Amplitude (cm)	0.66*	− 0.29

*P < 0.05; **P < 0.01; ***P < 0.001.

## Discussion

The gut-brain axis is increasingly investigated for its potential role in FP behaviour. While there is cursory evidence of the importance of the gut microbiota in FP^18,19,21,22^, the current body of literature lacks studies conducted under controlled settings and those employing a reductionist approach to assess foundational links between FP, gut function and the resident microbiota. Understanding this relationship has implications for developing novel tactics to circumvent or prevent FP in large flocks of laying hens, a significant welfare issue resulting in injury and mortality in farmed chicken. To address this knowledge gap, we adapted a mammalian ex-vivo organ bath to record cecal motility in domestic birds classified as peckers (P) and non-peckers (NP). This system was, then, used to examine the effect of a probiotic (*L. rhamnosus*) treatment on motility measurements.

We report the successful adaptation of an organ bath using lidocaine to help initiate ex-vivo contractions in ceca of laying hens. Contractions were recorded in over 96% of the subjects tested, attesting to the fidelity of the model presented herein. On average, approximately 62% of the ceca placed in the organ bath contracted, similarly to observations by Hodgkiss^41^ reporting contractions in 64% of ceca in the presence of tetrodotoxin. The data presented here further corroborates the findings in turkeys by Duke ^42^ where the two ceca of a bird exhibited similar motility measurements. We further conclude that given the comparability of measurements between the two ceca of individual birds, ceca can provide reliable measurements up to 1.5 h post-extraction, and that an accurate motility profile can be determined using a single cecum.

Avian GI motility is a neglected field of research^43^. Research published within the last decade involves young broilers^44^ and gut motility development in chicken embryos^45^^,^ while the most recent studies using laying hens date back to the 1990’s^41–43,46^. Our work, therefore, provides a critical update of adult laying hen gut motility and, most importantly, its relationship to FP behaviour. Indeed, this study represents the first ex-vivo motility measurements in P and NP birds and, as such, it contributes to the foundational knowledge base of cecal motility in birds classified as peckers. The ceca play an important role in birds as a site for digestion, fermentation, utilization and absorption of water and nitrogenous components, beneficial and pathogenic bacteria, and production of immunoglobulins and antibodies reviewed by Clench and Mathias^47^. It is, moreover, the site of greatest microbial density within the GI tract of chickens^48^. Consequently, it is conceivable that the ceca and its microbiota may directly or indirectly influence behaviour through the gut-brain axis or through its interaction with the ENS, making this component of the GI tract a valuable point of study when investigating contributing factors to FP behaviour.

This is also the first study to report the effect of a probiotic, such as *L. rhamnosus*, on cecal motility in laying hens. We found that *L. rhamnosus* decreased the frequency of cecal contractions by 68% and increased their amplitude by 58% in hens, regardless of FP phenotype. A similar trend was observed by others in stressed mice, whereby *L. rhamnosus* treatment decreased the frequency and amplitude of contractions in the colon and jejunum to varying degrees^35,40^, with the notable exception in West et al.^35^ who reported a deviation where the frequency of contractions in the jejenum increased by 88% upon exposure to the probiotic. Both groups also observed that the velocity of contractions was significantly altered in the presence of *L. rhamnosus*^35,40^, contradicting the data in the present study. These reports suggest that the effect of the probiotic on measures of gut motility in mice is dependent on the region tested^35,40^ (e.g., the response of ceca to *L. rhamnosus* has not been tested in mice), and the stressors to which the animals are exposed^35^. These variables may, then, also account for discrepancies between studies and they must also be considered in future laying hen research. Finally, it cannot be ignored that species-related differences are equally likely to play a role in distinct contraction profiles. It is tempting to speculate that the decrease in frequency combined with the tendency for increased amplitude of contractions observed in our data suggest less frequent, but stronger contractions to mix or empty the cecal content in the presence of *L. rhamnosus,* leading to faster turnover of contents and a generally faster feed passage time. It is noteworthy that lactobacilli are used to promote the frequency of bowel movements in human patients experiencing constipation^49,50^, however, the precise mechanism of action of probiotics on gut motility remains unclear^28^. Furthermore, probiotic bacteria, including strains of *Lactobacillus*, are known to modulate the activity of the ENS which exerts local control over mixing and propulsive movements of the intestine^31,51^. Interestingly, Grasa et al.^52^ found that mice whose microbiota were depleted through antibiotic treatment had a reduced amplitude of contractions in the ileum and colon. As such, it cannot be ignored that the increase in the amplitude of contractions in the presence of *L. rhamnosus* that was observed in the current study may be a generalized reaction to the presence of a bacterium in the lumen of the ceca. Further research is required to elucidate the mode of action that *L. rhamnosus* employs to change motility measures, understand its role as part of the gut microbiota and determine its interactions with other systems within the brain-gut-microbiota axis.

In addition to establishing the general impact of the probiotic, we further tested whether the potentially inherent differences in the brain-GI-microbiota axis components of P and NP birds resulted in different responses to the cecal *L. rhamnosus* treatment. It must be noted that, contrary to our initial hypothesis, the baseline cecal contractions between P and NP birds were similar. Moreover, the ceca of P and NP birds reacted similarly to the *L. rhamnosus* treatment. However, we report that the number of FP bouts performed by a bird correlated positively with the velocity and amplitude of contraction within the P bird population. No such correlation was observed within the NP group. Interestingly, birds from a high FP line are reported to have higher levels of locomotor activity compared to birds from a low FP line^11^ and altered feed passage time resulting in faster movement of ingesta through the GI tract^20^. The reason for this faster transit time is unclear, but changes to gut motility is a promising factor to consider and may explain the higher cecal motility measures for P birds observed in the current study. In support of this theory, evidence in mammals suggests that acute and chronic exercise increases colonic motility^53^. If extrapolated to the chicken model, one can postulate that the inherent increased physical activity of peckers causes faster feed passage time. Yet another theory suggests that higher feather consumption in P birds compared to NP birds influences the differences observed in gut motility^20,54,55^. Higher volumes of ingested feathers presumably increase gut motility in P birds by increasing contractions, which, in turn, results in the observed faster feed passage time. The positive correlation between the number of FP bouts performed and both the velocity and amplitude (possibly correlated with the strength of contractions) of cecal contractions observed in the current study could support this hypothesis. Future research would need to establish links between the level of locomotor activity, feather consumption, FP and cecal contractions using the model presented in this work.

Previous studies have established associations between FP, microbiota profiles and microbial metabolism^18,19,21,22^. Most notably, Birkl et al.^18^ and van der Eijk et al.^19^ reported a lower abundance of *Lactobacillus* spp. in P birds compared to NP birds. In mice, *L. rhamnosus* has anxiolytic effects; reducing anxiety and depression-like behaviour, while increasing exploratory behaviour^33^. This highlights its potential to influence brain chemistry and behaviour possibly by acting via ENS^32,33^.

Furthermore, *Lactobacillus* strains can influence tryptophan metabolism, as well as the kynurenine and serotonergic pathways which are involved in brain-gut disorders^56^. Importantly, these are candidate pathways suspected of contributing to FP^12^. Our data establishes that *L. rhamnosus* increases contraction measures regardless of pecking phenotype. It must also be considered that, while P and NP ceca responded similarly to *L. rhamnosus* under the conditions tested, the probiotic may elicit a significantly higher response in live P subjects as they naturally have a low abundance of *Lactobacilli*. Furthermore, we found a first indication that FP behaviour is correlated with measures of gut motility in P birds. Taken together with the findings of previous groups^12,18,33,56^, this data offers the exciting possibility of controlling FP behaviour in laying hens by manipulating the cecal microbiota to increase the abundance of *Lactobacilli*. Extensive future investigations are required to establish a feedback loop between *L. rhamnosus* supplementation and FP, and to confirm whether the probiotic can be employed as a preventive or curative therapy for FP behaviour.

Consequently, we are further investigating the impact of a *L. rhamnosus* treatment on FP behaviour as part of another study. Interestingly, early-life transplantation of microbiota of P birds, with a low abundance of *Lactobacillales,* into NP birds and vice versa found little impact on FP behaviour during the first 15 weeks of life^57^. Nevertheless, it was observed that altering the microbiota caused changes in birds’ fearfulness levels, immune characteristics, and peripheral serotonin levels^57^. It is important to recognize that these studies do not allow researchers to tease apart modes of action of individual microbiota and how it affects behaviour of individual systems in an interrelated brain-GI-microbiota axis. A combination of fundamental research that elucidate the impact of probiotics on gut motility, and interactions between gut motility and the brain-GI-microbiota axis, together with applied research to identify effects on behaviour are required to develop successful and viable strategies to manage FP in the millions of domesticated chickens used for food production.

In conclusion, this research demonstrates the successful adaptation of a mammalian ex-vivo organ perfusion system to measure gut motility in an avian species addressing a neglected field of research in avian physiology. We measured velocity, frequency and amplitude of cecal contractions in laying hens in the presence of a single probiotic (*L. rhamnosus JB-1*) as an intraluminal stimulus and correlated these measurements to differences in feather pecking behaviour. The presence of *L. rhamnosus* reduced frequency and tended to increase amplitude of cecal contractions regardless of FP behaviour, showing the potential of probiotics to target gut motility. While no differences in velocity, frequency or amplitude of cecal contractions were observed between peckers and non-peckers, the velocity and amplitude of contractions were positively correlated with the number of FP bouts in peckers. This suggests that gut motility and FP behaviour are linked, though the direction of this relationship remains unclear. The results presented in this study open a new avenue of research to address fundamental questions regarding behaviour and gastro-intestinal function in laying hens by targeting the brain-GI-microbiota axis. Further, the potential for *Lactobacillus* spp. to act as a corrective therapy for this disruptive behaviour is exciting and warrants further investigation.

## Methods

### Animals and housing

We used 120 non-beak trimmed female chickens (White Leghorn) originating from a genetic line selected for high (HFP) levels of FP behaviour and an unselected control (CON) line^58^. From hatch, birds were housed in enriched floor pens (5 HFP and 5 CON birds/pen, 118 × 118 cm) equipped with a feeder, drinker, wood shavings, elevated perches and a nest box at the University of Guelph. One security camera (Samsung SNO-5080R, IR, Samsung Techwin CO., Gyeongi-do Korea) was mounted at the top of each pen. Feed and water were provided ad libitum and birds were kept under natural daylight conditions.

Bird behaviour was recorded to determine the amount of FP performed by each bird at 52 weeks of age. Birds were individually identified ten days prior to start of the recordings by fastening numbered silicone plates to the backs of the birds using elastic straps around their wings^59^. We recorded behaviour two days per week between 10:00 and 14:00 h over a 6-week period. All occurrences of severe FP were recorded during 10-min observation periods (one in the morning and one in the afternoon) and averaged to determine the number of FP bouts performed by individual birds^60–62^.

In order to investigate the relationship between FP and cecal motility, we continued selection from the genetic lines by using birds with extreme pecker (P) and non-pecker (NP) phenotypes. We chose 13 P (9 birds from the HFP and 4 birds from the CON line) and 17 NP (7 birds from the HFP and 10 birds from the CON line) birds which showed a significantly different average number of FP bouts per day (P: 12.3 ± 2.14 vs NP: 1.0 ± 0.16, F_1,23_ = 113.47, *P* < 0.001).

### Cecal motility recordings: treatments

**Figure 1:**
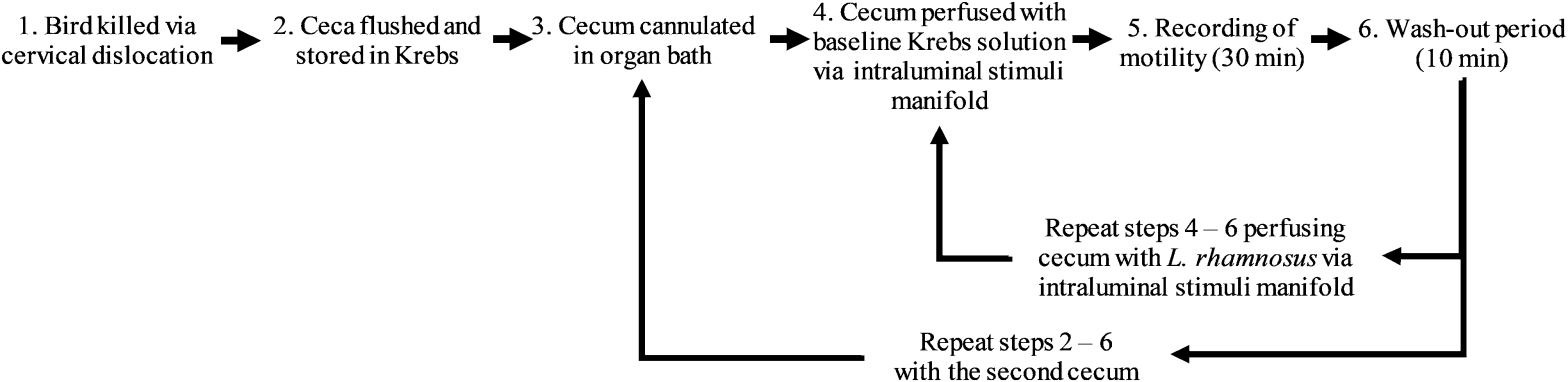
Schematic representation of organ bath set-up and procedure.

The 13 P and 17 NP birds were killed by cervical dislocation to conduct cecal motility recordings in an organ bath system (Fig. 1) in a similar manner as described in West et al.^35^. Following cervical dislocation, ceca of the bird were removed at the junction of the ileum with the colon and placed in a beaker of fresh Krebs buffer solution (mmol/L: 118 NaCl, 4.8 KCl, 25 NaHCO_3_, 1.0 NaH_2_PO_4_, 1.2 MgSO_4_, 11.1 glucose, and 2.5 CaCl_2_ (Sigma-Aldrich, 2017), and continuously bubbled with carbogen gas (95% O_2_ and 5% CO_2_) to ensure viability of the tissue^35^. Ceca were separated and any excess mesentery and fat tissue attached to each cecum was removed. A 0.5 cm incision was made at the caudal end of the tissue and any remaining digesta flushed out of the ceca with Krebs.

The cecum was placed in the well of a heated organ bath perfusion system filled with the same oxygenated Krebs buffer solution. Organ bath temperature was maintained at 38 °C using an external heat exchanger^41^. The cranial and caudal end of the cecum were cannulated with silicone tubing. This tubing was attached to a manifold at the cranial end to allow continuous intraluminal inflow of treatment solutions, and which drained from the caudal end into an outflow beaker outside of the organ bath system^35^. Two intraluminal stimuli were used during the experiment: (1) baseline Krebs solution (hereafter referred to as ‘baseline treatment’) and (2) *Lactobacillus rhamnosus (JB-1)* diluted in Krebs solution (hereafter referred to as ‘*L. rhamnosus* treatment’). *Lactobacillus rhamnosus (JB-1)* came from an in-house stock at McMaster University provided by Alimentary Health Inc., Cork, Ireland and was diluted with Krebs solution to concentrations of 1 × 10E8 CFU/ml as described in West et al.^35^. The manifold set-up and 50 mL plastic syringe tubes attached to it allowed different intraluminal treatment solutions to be applied intraluminally by opening or closing individual stopcocks of the manifold. The manifold was raised above the level of the tissue such that the intraluminal pressure in the tissue was just sufficient to stimulate contractile activity^35^. In contrast to mammalian tissue, avian intestinal tissues require additional specific stimulation for contractions to be initiated^41^ and, therefore, we used lidocaine hydrochloride monohydrate (Sigma-Aldrich, 2017) as a local anaesthetic to elicit peristalsis. Lidocaine hydrochloride monohydrate was dissolved in Krebs solution and added directly to the organ bath at a concentration of 375 µg/ml 1–2 min before the start of each recording^41^.

A video camera (Microsoft LifeCam HD-3000) was mounted 15 cm above the bath to record cecal motility. Recordings were conducted for 30 min during perfusion of each of the two intraluminal stimuli treatments. A 10 min wash-out period with Krebs solution was observed between treatments. As such, each cecum was in the organ bath for approx. 1.5 h (including preparation and 2 × 30 min recording time, see Fig. 1) meaning that the full process lasted approx. 3 h per bird.

### Cecal motility recordings: video analysis

**Figure 2:**
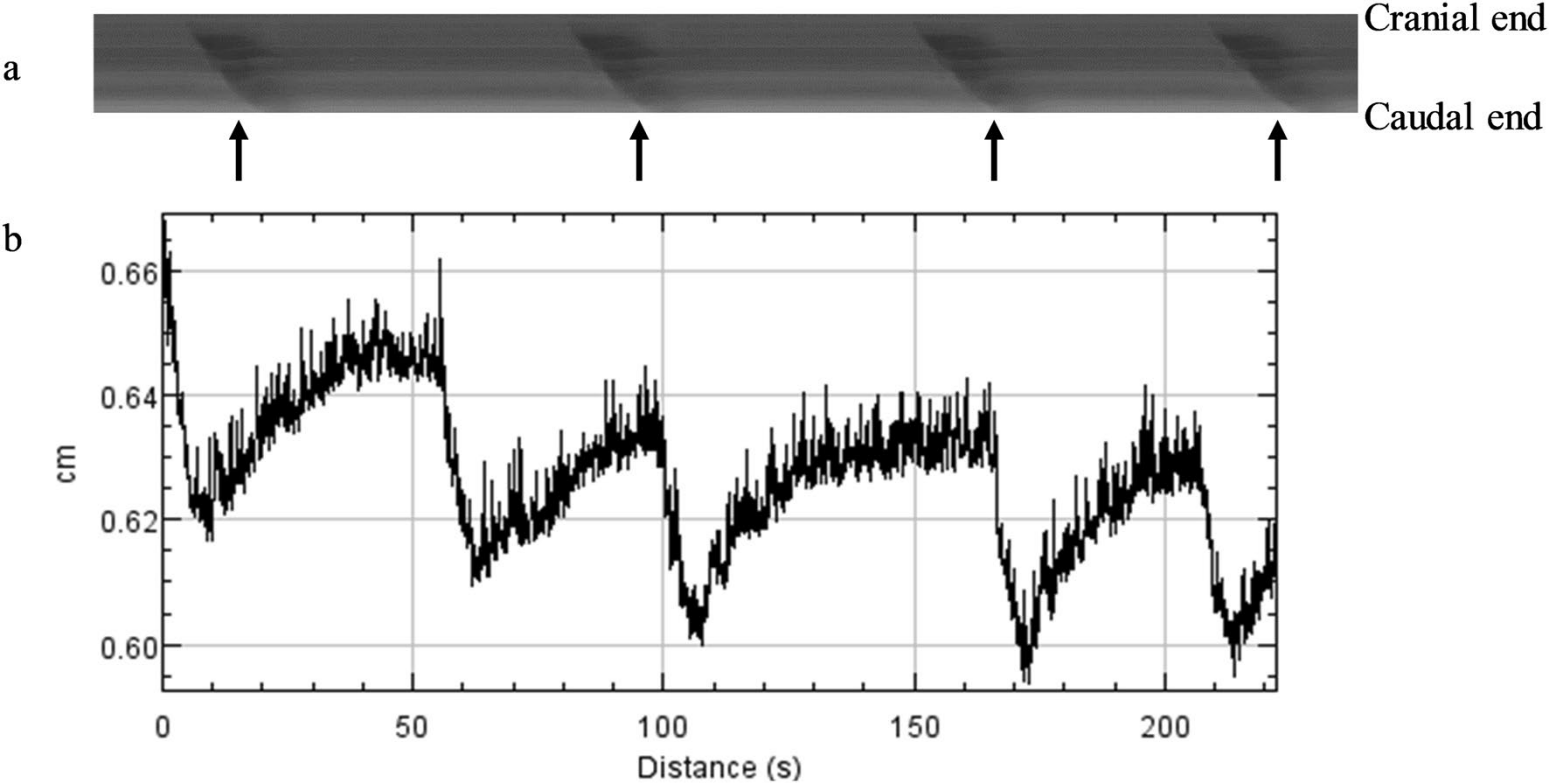
Example of spatiotemporal maps generated through the ImageJ software (version 1.51q; NIH, Bethesda, MD, USA, 2017) with the DMapLE plug-in (developed by Dr. Sean Parsons and freely available at https://scepticalphysiologist.com/code/code.html) for recordings of cecal contractions in laying hens. Maps display the diameter across the cecum from the cranial to caudal end vertically (cm) and over time (s) horizontally. The diameter of the gut is displayed as bands of light (relaxation; larger diameter) and dark (contraction; smaller diameter) hues (**a**). In the current example, four contractions running from the cranial to the caudal end can be observed (indicated with arrows). Velocity is determined by measuring the slope of contraction, frequency as the number of contractions within a given time period, and amplitude as the height of the diameter change at peak contraction (**a**). Note that these are examples and therefore the bands in a do not correspond to the peaks in (**b**).

Video recordings were transferred to the VideoPad Video Editor (version 5.20; NHC Software, Greenwood Village, CO, USA, 2017) and analyzed using ImageJ software (version 1.51q; NIH, Bethesda, MD, USA, 2017) with a specific plug-in (DMapLE; developed by Dr. Sean Parsons and freely available at https://scepticalphysiologist.com/code/code.html) in order to generate spatiotemporal maps as described by Wu et al.^40^. Spatiotemporal maps depict the contractility of tissue over time and enable the measurement of previously validated motility parameters i.e., velocity, frequency and amplitude of contractions^30,35,40^. Using an edge detection algorithm, the diameter across the cecum is displayed as bands of light (relaxation, larger diameter) and dark (contraction, smaller diameter) hues (Fig. 2). The maps run from the cranial to caudal end (Y-axis, cm) and across time (X-axis, s). Velocity, frequency and amplitude of contractions were determined as per West et al.^37^. Velocity (cm/s) of cecal contractions was calculated by averaging the slope of propagating contractions (Fig. 2). Frequency (Hz) of contractions was determined by the number of contractions within a given time interval (Fig. 2). Amplitude (cm) of contractions was determined by averaging the change in cecal diameter during peak contractions (Fig. 2).

### Statistical analysis

All statistical procedures were conducted using SAS V9.4 (SAS Inst. Inc., Cary, NC). The assumptions of normally distributed residuals and homogeneity of variance were examined graphically with the use of QQ plots. Data were transformed where necessary. Statistical significance was considered at *P* < 0.05 and tendencies are reported when 0.05 ≤ *P* ≤ 0.1. Values are presented as LS means ± SE, unless stated otherwise.

One bird did not show any cecal contractions during both the baseline and *L. rhamnosus* treatment and was excluded from analysis. First, we investigated if there was a difference between the two ceca of each bird in terms of velocity, frequency and amplitude of contractions during the baseline treatment using generalized linear mixed models with cecum number as a fixed effect. No differences were found and, therefore, values for these parameters were averaged in the final dataset for birds where both ceca showed contractions in the organ bath; for those birds where contractions were only observed in one cecum in the organ bath, this value was used.

Due to the limited number of birds available for testing, we chose to focus on differences in phenotypic FP profiles as a continuation of genetic selection. The effect of FP phenotype (NP, P), treatment (Baseline, *L. rhamnosus*) and their interaction on cecal motility as expressed by velocity, frequency and amplitude was examined using generalized linear mixed models with a repeated measure autoregressive covariance structure. A Tukey–Kramer adjustment was used to account for multiple comparisons.

Relationships between FP and cecal contractions were explored further within the baseline treatment only to describe intrinsic relationships, and avoid any possible confounding with the *L. rhamnosus* treatment. Spearman rank correlations were calculated between the average number of FP bouts performed and the velocity, frequency, and amplitude of contractions within each phenotypic class.

### Ethical approval

This study was approved by the University of Guelph Animal Care Committee (Animal Utilization Protocol Number 3206). The study was carried out in accordance with relevant guidelines and regulations.

## Data Availability

The datasets generated during and/or analysed during the current study are available from the corresponding author on reasonable request.

## References

[1] Rubinstein J, Lightfoot T (2014). Feather loss and feather destructive behavior in pet birds. Vet. Clin. N. Am. Exot. Anim. Pract..

[2] Gaskins LA, Bergman L (2011). Surveys of avian practitioners and pet owners regarding common behavior problems in psittacine birds. J. Avian Med. Surg..

[3] Rodenburg TB (2013). The prevention and control of feather pecking in laying hens: identifying the underlying principles. Worlds Poult. Sci. J..

[4] Nicol CJ (2013). The prevention and control of feather pecking: application to commercial systems. Worlds Poult. Sci. J..

[5] Elkhoraibi C, Blatchford RA, Pitesky ME, Mench JA (2014). Backyard chickens in the United States: a survey of flock owners. Poult. Sci..

[6] van Staaveren N, Harlander-Matauschek A, Nicol C (2020). Chapter 16: Cause and prevention of injurious pecking in poultry. Understanding the behaviour and improving the welfare of chickens.

[7] Green LE, Lewis K, Kimpton A, Nicol CJ (2000). Cross-sectional study of the prevalence of feather pecking in laying hens in alternative systems and its associations with management and disease. Vet. Rec..

[8] Bestman M, Wagenaar J-P (2014). Health and welfare in Dutch organic laying hens. Animals.

[9] de Haas EN, Kemp B, Bolhuis JE, Groothuis T, Rodenburg TB (2013). Fear, stress, and feather pecking in commercial white and brown laying hen parent-stock flocks and their relationships with production parameters. Poult. Sci..

[10] Kjaer JB, Guémené D (2009). Adrenal reactivity in lines of domestic fowl selected on feather pecking behavior. Physiol. Behav..

[11] Kjaer JB (2009). Feather pecking in domestic fowl is genetically related to locomotor activity levels: implications for a hyperactivity disorder model of feather pecking. Behav. Genet..

[12] de Haas EN, van der Eijk JAJ (2018). Where in the serotonergic system does it go wrong? Unravelling the route by which the serotonergic system affects feather pecking in chickens. Neurosci. Biobehav. Rev..

[13] Van Hierden YM (2002). Adrenocortical reactivity and central serotonin and dopamine turnover in young chicks from a high and low feather-pecking line of laying hens. Physiol. Behav..

[14] Birkl P (2019). The role of tryptophan-kynurenine in feather pecking in domestic chicken lines. Front. Vet. Sci..

[15] Kops MS (2017). Brain monoamine levels and behaviour of young and adult chickens genetically selected on feather pecking. Behav. Brain Res..

[16] Kjaer JB, Hjarvard BM, Jensen KH, Hansen-Møller J, Naesbye Larsen O (2004). Effects of haloperidol, a dopamine D2 receptor antagonist, on feather pecking behaviour in laying hens. Appl. Anim. Behav. Sci..

[17] Brunberg EI (2016). Omnivores going astray: a review and new synthesis of abnormal behavior in pigs and laying hens. Front. Vet. Sci..

[18] Birkl P (2018). Differences in cecal microbiome of selected high and low feather-pecking laying hens. Poult. Sci..

[19] van der Eijk JAJ (2019). Differences in gut microbiota composition of laying hen lines divergently selected on feather pecking. Poult. Sci..

[20] Harlander-Matauschek A, Piepho HP, Bessei W (2006). The effect of feather eating on feed passage in laying hens. Poult. Sci..

[21] Meyer B, Bessei AW, Vahjen W, Zentek J, Harlander-Matauschek A (2012). Dietary inclusion of feathers affects intestinal microbiota and microbial metabolites in growing Leghorn-type chickens. Poult. Sci..

[22] Meyer B, Zentek J, Harlander-Matauschek A (2013). Differences in intestinal microbial metabolites in laying hens with high and low levels of repetitive feather-pecking behavior. Physiol. Behav..

[23] Cenit MC, Nuevo IC, Codoñer-Franch P, Dinan TG, Sanz Y (2017). Gut microbiota and attention deficit hyperactivity disorder: new perspectives for a challenging condition. Eur. Child Adolesc. Psychiatry.

[24] Dash S, Clarke G, Berk M, Jacka FN (2015). The gut microbiome and diet in psychiatry: focus on depression. Curr. Opin. Psychiatry.

[25] Mulle JG, Sharp WG, Cubells JF (2013). The gut microbiome: a new frontier in autism research. Curr. Psychiatry Rep..

[26] McElhanon BO, McCracken C, Karpen S, Sharp WG (2014). Gastrointestinal symptoms in autism spectrum disorder: a meta-analysis. Pediatrics.

[27] van Sadelhoff JHJ (2019). The gut-immune-brain axis in autism spectrum disorders; a focus on amino acids. Front. Endocrinol. (Lausanne).

[28] Dimidi E, Christodoulides S, Scott SM, Whelan K (2017). Mechanisms of action of probiotics and the gastrointestinal microbiota on gut motility and constipation. Adv. Nutr..

[29] Margolis KG (2016). Serotonin transporter variant drives preventable gastrointestinal abnormalities in development and function. J. Clin. Invest..

[30] Wang B (2010). Luminal administration ex vivo of a live Lactobacillus species moderates mouse jejunal motility within minutes. FASEB J..

[31] Kunze WA (2009). *Lactobacillus reuteri* enhances excitability of colonic AH neurons by inhibiting calcium-dependent potassium channel opening. J. Cell. Mol. Med..

[32] Perez-Burgos A, Mao Y-K, Bienenstock J, Kunze WA (2014). The gut-brain axis rewired: adding a functional vagal nicotinic “sensory synapse”. FASEB J..

[33] Bravo JA (2011). Ingestion of Lactobacillus strain regulates emotional behavior and central GABA receptor expression in a mouse via the vagus nerve. Proc. Natl. Acad. Sci. U. S. A..

[34] Forsythe P, Kunze WA (2013). Voices from within: gut microbes and the CNS. Cell. Mol. Life Sci..

[35] West C (2017). Lactobacillus rhamnosus strain JB-1 reverses restraint stress-induced gut dysmotility. Neurogastroenterol. Motil..

[36] West C, Stanisz AM, Wong A, Kunze WA (2016). Effects of Saccharomyces cerevisiae or boulardii yeasts on acute stress induced intestinal dysmotility. World J. Gastroenterol..

[37] West CL (2019). Colonic motility and jejunal vagal afferent firing rates are decreased in aged adult male mice and can be restored by an aminosterol. Front. Neurosci..

[38] McFarland LV (2010). Systematic review and meta-analysis of Saccharomyces boulardii in adult patients. World J. Gastroenterol..

[39] Mohd Shaufi MA, Sieo CC, Chong CW, Gan HM, Ho YW (2015). Deciphering chicken gut microbial dynamics based on high-throughput 16S rRNA metagenomics analyses. Gut Pathog..

[40] Wu RY (2013). Spatiotemporal maps reveal regional differences in the effects on gut motility for *Lactobacillus reuteri* and *rhamnosus* strains. Neurogastroenterol. Motil..

[41] Hodgkiss JP (1984). Peristalsis and antiperistalsis in the chicken caecum are myogenic. Q. J. Exp. Physiol..

[42] Duke GE (1989). Relationship of cecal and colonic motility to diet, habitat, and cecal anatomy in several avian species. J. Exp. Zool. Suppl..

[43] Duke GE (1982). Gastrointestinal motility and its regulation. Poult. Sci..

[44] Janssen PWM, Lentle RG, Hulls C, Ravindran V, Amerah AM (2009). Spatiotemporal mapping of the motility of the isolated chicken caecum. J. Comp. Physiol. B Biochem. Syst. Environ. Physiol..

[45] Chevalier NR, Fleury V, Dufour S, Proux-Gillardeaux V, Asnacios A (2017). Emergence and development of gut motility in the chicken embryo. PLoS ONE.

[46] Rawson RE, Duke GE, Brown DR (1990). Effect of avian neurotensin on motility of chicken (Gallus domesticus) lower gut in vivo and in vitro. Peptides.

[47] Clench MH, Mathias JR (1995). The avian cecum: a review. Wilson Bull..

[48] Oakley BB (2014). The chicken gastrointestinal microbiome. FEMS Microbiol. Lett..

[49] Del Piano M (2010). The use of probiotics in healthy volunteers with evacuation disorders and hard stools: a double-blind, randomized, placebo-controlled study. J. Clin. Gastroenterol..

[50] Ojetti V (2014). The effect of *Lactobacillus reuteri* supplementation in adults with chronic functional constipation: a randomized, double-blind, placebo-controlled trial. J. Gastrointestin. Liver Dis..

[51] Kunze WA, Furness JB (1999). The enteric nervous system and regulation of intestinal motility. Annu. Rev. Physiol..

[52] Grasa L (2015). Antibiotic-induced depletion of murine microbiota induces mild inflammation and changes in toll-like receptor patterns and intestinal motility. Microb. Ecol..

[53] Gisolfi CV (2000). Is the GI system built for exercise?. News Physiol. Sci..

[54] McKeegan DE, Savory C (2001). Feather eating in individually caged hens which differ in their propensity to feather peck. Appl. Anim. Behav. Sci..

[55] McKeegan DEF, Savory CJ (1999). Feather eating in layer pullets and its possible role in the aetiology of feather pecking damage. Appl. Anim. Behav. Sci..

[56] O’Mahony SM, Clarke G, Borre YE, Dinan TG, Cryan JF (2015). Serotonin, tryptophan metabolism and the brain-gut-microbiome axis. Behav. Brain Res..

[57] van der Eijk JAJ (2020). Early-life microbiota transplantation affects behavioural responses, serotonin and immune characteristics in chicken lines divergently selected on feather pecking. Sci. Rep..

[58] Kjaer JB, Sørensen P, Su G (2001). Divergent selection on feather pecking behaviour in laying hens (*Gallus gallus domesticus*). Appl. Anim. Behav. Sci..

[59] Harlander Matauschek A, Beck P, Rodenburg TB (2010). Effect of an early bitter taste experience on subsequent feather-pecking behaviour in laying hens. Appl. Anim. Behav. Sci..

[60] Altmann J (1974). Observational study of behavior: sampling methods. Behaviour.

[61] Bilčik B, Keeling LJ (2000). Relationship between feather pecking and ground pecking in laying hens and the effect of group size. Appl. Anim. Behav. Sci..

[62] Zeltner E, Klein T, Huber-Eicher B (2000). Is there social transmission of feather pecking in groups of laying hen chicks?. Anim. Behav..

